# Doxycycline and eradication of microfilaremia in patients with loiasis.

**DOI:** 10.3201/eid0707.010747

**Published:** 2001

**Authors:** P Brouqui, P E Fournier, D Raoult


2. World Health Organization. Control of the leishmaniases. Report of a WHO Expert Committee. World Health Organ Tech Rep Ser 1990;793:1-158.
3. El Harith A, Kolk AH, Kager PA, Leeuwenburg J, Muigai R, Kiugu S, et al. A simple and economical direct agglutination test for serodiagnosis and sero-epidemiological studies of visceral leishmaniasis. Trans R Soc Trop Med Hyg 1986;80:583-7.
4. Shiddo SA, Aden Mohamed A, Akuffo HO, Mohamud KA, Herzi AA, Herzi Mohamed H, et al. Visceral leishmaniasis in Somalia: prevalence of markers of infection and disease manifestations in a village in an endemic area. Trans R Soc Trop Med Hyg 1995;89:361-5.
5. Woolhead A. A recent case of visceral leishmaniasis in Somalia. Ann Trop Med Parasitol 1995;89:687-8.
6. Cerf BJ, Jones TC, Badaro R, Sampaio D, Teixeira R, Johnson WD Jr. Malnutrition as a risk factor for severe visceral leishmaniasis. J Infect Dis 1987;156:1030-3.
7. Seaman J, Mercer AJ, Sondorp E. The epidemic of visceral leishmaniasis in western Upper Nile, southern Sudan: course and impact from 1984 to 1994. Int J Epidemiol 1996;25:862-871.
8. Boelaert M, Englebert M, Hanquet G, Van Damme W, Van der Stuyft P. Refugee relief rations. Lancet 1997;349:1775.

### Doxycycline and Eradication of Microfilaremia in Patients with Loiasis

**To the Editor:** *Wolbachia* are intracellular symbionts found in 20% of insects and in several nematodes, including filarial worms. Because tetracycline eradicates *Wolbachia* in nematodes, this drug has been proposed for chemotherapy in filariasis (1). We report two patients with loiasis in whom no Wolbachial DNA was detected in microfilariae by polymerase chain reaction (PCR), and for whom 6 weeks of doxycycline failed to eradicate the microfilaremia. We conclude that doxycycline may not be an efficient therapy for loiasis.

Filariae are responsible for 150 million infections worldwide, some of them devastating diseases such as elephantiasis (caused by Bancroftian and Brugian filariasis) and blindness (caused by onchocerciasis). There is no satisfactory treatment for filariasis: although diethycarbamazine citrate has been used for 50 years to treat the disease, this drug is not efficient in most adults (2). Ivermectin has been reported to be efficient in treating microfilaremia and possibly for viability and fertility of adult *Oncocerca volvulus* worms if therapy is prolonged for 2 to 3 years (2). However, the microfilaricidal effect of ivermectin on *Wuchereria*, *Brugia*, and *Loa loa* is similar to diethycarbamazine: ultrasonography shows that it has no effect on adult Bancroftian worms, and microfilariae often reappear after a few months (2).

Intracellular bacteria have been observed in the lateral cords of adult female worms as well as in microfilariae of *W. bancrofti,* and these bacteria have recently been identified as belonging to the *Wolbachia* genogroup (3). *Wolbachia* have been detected by PCR in Brugian and Bancroftian filariae, dirofilariae, and most species of *Oncocerca* including *O. volvulus*, but have never been detected in *L. loa* worms (4). *Wolbachia* are causative agents of a variety of modifications in host development and reproduction, including cytoplasmic incompatibility and parthenogenesis. Consequently, it has been proposed that antibiotic eradication of *Wolbachia* from infected filarial worms would reduce microfilaremia. This has been demonstrated in an animal model with *Litomosoides sigmodontis* and recently confirmed in patients infected with *O. volvulus* (1-5). In experimental *L. sigmodontis* infection, after 41 days of therapy, microfilaremia in tetracycline-treated animals was one-tenth that in normally infected animals (5).

We report the failure of tetracycline to reduce microfilaremia in two patients with *L. loa* filariasis. Patient no. 1 was a 58-year-old man who worked in Gabon for many years and had cutaneous larva migrans. *L. loa* microfilaremia was detected ($4x10^3$/mL). Patient no. 2, a 15-year-old boy living in Cameroon, was diagnosed with Calabar swelling with *L. loa* microfilaremia ($1x10^3$/mL). After giving informed consent, both patients were treated with doxycycline 200 mg daily for 6 weeks as previously described in *O. volvulus*-infected patients (1). We observed patients for microfilaremia every week for 6 weeks and then every 2 weeks for 2 months. The presence of adult worms was detected by physical examinations.

Microfilaremia was detected in both patients at the completion of treatment and at day 120 of follow-up. In patient no. 1, the frequency of migrating adult worms seemed to diminish during therapy, but they never disappeared. For *Wolbachia* detection in worms, blood samples were collected both in Dupont-Isolator and EDTA-containing tubes. After centrifugation at 5000 x *g* for 30 min, the worm-enriched pellet was resuspended in 1 mL of sterile deionized water for erythrocyte lysis. DNA was extracted from the suspension by using the QIAmp-blood kit (Qiagen, Hilden, Germany) following manufacturer's recommendations. *W. pipientis* DNA was used as a positive control. Control of DNA extraction was performed by amplifying microfilarial DNA using the nematode-specific 18S r DNA-derived primers 18SF (5'-GAT-ACC-GCC-CTA-GTT-CTG-ACC-3') and 18SR (5'-ACC-AAC-TAA-GAA-CGG-CCA-TG-3'). *Wolbachia* detection was attempted with the FD1 (5'-AGA-GTT-TGA-TCC-TGG-CTC-AG-3') and Rp2 (5'-ACG-GCT-ACC-TTG-TTA-CGA-CTT-3') eubacterial primers, with the *Ehrlichia* genus-specific 16S rDNA primers EHR16SD (5'-GGT-ACC-YAC-AGA-AGA-AGT-CC-3') and EHR16SR (5'- TAG-CAC-TCA-TCG-TTT-ACA-GC-3'), and with primers specific for the 16S r DNA of *B. malayi* endosymbiont, Bsymbf (5'-ACG-AGT-TAT-AGT-ATA-ACT-3'), and Bsymbr (5'-CCT-TCG-AAT-AGG-AAT-AAT-3') (3-6). PCR reactions were performed on PTC-200 thermocycler (MJ-Research, USA) by using 45 cycles of denaturation at 94°C for 30 sec, hybridization for 45 sec, and elongation at 72°C for 1 min. Hybridization temperatures were 55°C for FD1/Rp2, 53°C for EHR16SD/ EHR16SR, 42°C for Bsymbf/Bsymbr, and 57°C for 18SF/18SR. Experiments were repeated three times.

We detected *Wolbachia* in the positive control and 18s rRNA of the nematode in the sample, but no signal compatible with Wolbachial DNA was obtained with the sets of primers used. In fact, four species of filariae *(Dipetalonema setariosum*, *Acanthocheilonema vitae*, *O. flexuosa*, and *L. loa*) tested for intracellular bacteria by electron microscopy, immunohistochemistry, or PCR had no bacteria (4). The absence of *Wolbachia* in *L. loa* microfilariae may explain the failure of tetracycline therapy in our patients.

More work is needed to determine the prevalence of *Wolbachia* in filariae, their impact on fertility in each species, and the use of antibacterial agents for eradicating these pathogens.

### Acknowledgment

We thank MJ Taylor for providing *W. pipientis* DNA for controls.

**Philippe Brouqui, Pierre Edouard Fournier, and Didier Raoult**
Service des Maladies Infectieuses et Tropicales CHU Hôpital Nord et Unité des Rickettsies, Faculté de Médecine, Marseille, France

### References

1. Hoerauf A, Volkmann L, Hamelmann C, Adjei O, Autenrieth B, et al. Endosymbiotic bacteria in worms as targets for a novel chemotherapy in filariasis [Letter]. Lancet 2000;355:1242-3.
2. Grove DI. Tissue nematodes (trichinosis, dracunculiasis, filariasis). In: Mandel GJ, Bennett JF, Mandell DR, Douglas RG. Principles and practice of infectious diseases. Philadelphia: Churchill Livingstone; 2000. p. 22943-50.
3. Taylor MJ, Bilo K, Cross HF, Archer JP, Underwood AP. 16S rDNA phylogeny and ultrastructural characterization of *Wolbachia* intracellular bacteria of the filarial nematodes *Brugia malayi*, *B. pahangi*, and *Wuchereria bancrofti*. Exp Parasitol 1999;91:356-61.
4. Taylor MJ, Hoerauf A. *Wolbachia* bacteria of filarial nematodes. Parasitol Today 1999;15:437-42.
5. Hoerauf A, Nissen-Pahle K, Schmetz C, Henkle-Duhrsen K, Blaxter ML, et al. Tetracycline therapy targets intracellular bacteria in the filarial nematode *Litomosoides sigmodontis* and results in filarial infertility. J Clin Invest 1999;103:11-18.
6. Inokuma H, Raoult D, Brouqui P. Detection of *ehrlichia platys* DNA in brown dog ticks (*Rhipicephalus sanguineus*) in Okinawa Island, Japan. J Clin Microbiol 2000;38:4219-21.

### Bovine Spongiform Encephalopathy and Variant Creutzfeldt-Jakob Disease

**To the Editor:** The article by Brown et al. (1) contains the statement “it appears likely that changes in the rendering process that had taken place around 1980 allowed the etiologic agent in infected carcasses to survive.” If that is the case, why not revert to the rendering methods used before 1980? That measure would seem more cost-effective than trade embargoes and mass killing of cattle. Meal made from meat and bone was used as a livestock feed additive in many countries without the apparent disastrous effect seen in the United Kingdom. Did the rendering methods remain unchanged in these other countries? Historically, rendering was viewed somewhat differently in continental Europe; the primary purpose was not to make animal feed but to destroy infectious agents, as indicated by the very name of the facility: destruction plant (Destruktionsanstalt). The plants were under governmental inspection and there were mandatory time-temperature requirements for processing meat, bones, and other offal. Temperature requirements varied from 120°C to 140°C, which presumably would reduce if not eliminate prions. It would be of interest to see a description and objective analysis of rendering methods and regulations in various countries before and after 1980. In these times when the concept of hazard analysis and critical control points (HACCP) is gaining popularity, it would seem natural to extend the principle of process control to rendering. One might easily get the impression that policies to control bovine spongiform encephalitis are dominated by some of the stakeholders: the researchers writing their next research grant proposals, the public agencies eager to show prompt response, and those who would like to see their competitors’ beef kept off the market.

**Hans Riemann**
Department of Population Health and Reproduction
University of California, Davis, CA 95616 USA

### Reference

1. Brown P, Will RG, Bradley R, Asher DM, Detwiler L. Bovine spongiform encephalopathy and variant Creutzfeldt-Jakob disease: background, evolution, and current concerns. Emerg Infect Dis 2001;7:6-16.

### Mad Cow Disease

**To the Editor:** In regards to “Bovine Spongiform Encephalopathy and  Variant Creutzfeldt-Jakob Disease: Background, Evolution, and Current Concerns” (1), use of the name “variant Creutzfeldt-Jacob disease” (vCJD) for human cases of bovine spongiform encephalopathy (BSE) is regrettable. The disease that occurs in humans exposed to BSE is zoonotic and CJD is not; in addition, the human form of BSE has important clinical, pathologic, and epidemiologic differences from CJD. Continued use of this terminology perpetuates the error.

The fact that 12 years after the feed ban bovine cases continue to occur in the United Kingdom at a much higher rate than in any other country could have two possible causes: inefficient controls or additional routes of transmission. Data on alternative routes of transmission must be evaluated, and important gaps in our understanding of BSE in cattle must be addressed.

Extensive epidemiologic data on BSE in the United Kingdom seemed to clearly implicate the practice of feeding cattle bovine offal as the primary, if not the sole, cause of the spread of BSE. Alternative theories for the origin and spread of BSE, e.g., use of insecticides on bovines or the practice of artificial insemination, appear to have been ruled out quickly on the basis of early epidemiologic data. Confidence in the reliability of these data seems to have been so great as to unduly delay transmission experiments to assess the role of alternative pathways (e.g., artificial insemination) in the propagation of BSE. Is prion protein present in semen or is it not? What if it were present in semen? Would this route lead to shorter incubation than the alimentary route? There seems to be no experimental information on the effect of freezing mutated prion protein to -196°C and placing it, after thawing, directly on the stimulated uterine mucosa.

Although the United Kingdom has acknowledged that compliance with the feed ban improved after 1996, we should not be too eager to accept lack of compliance as the only possible reason for the persistence of BSE. Fifteen years after the BSE epidemic began, it cannot be disputed that the ban on offal feed has interrupted spread of the disease. However, if continued spread by the alimentary route can be excluded as the cause of more recent cases, each of these cases should be carefully evaluated to uncover heretofore unknown or underappreciated routes of transmission.

**Werner Slenczka**
Klinikum der Philipps-Universität Marburg, Marburg, Germany

### Reference

1. Brown P, Will RG, Bradley R, Asher DM, Detwiler L. Bovine spongiform encephalopathy and variant Creutzfeldt-Jakob disease: background, evolution, and current concerns. Emerg Infect Dis 2001;7:6-14.

